# The Long Noncoding RNA *HEAL* Regulates HIV-1 Replication through Epigenetic Regulation of the HIV-1 Promoter

**DOI:** 10.1128/mBio.02016-19

**Published:** 2019-09-24

**Authors:** Ti-Chun Chao, Qiong Zhang, Zhonghan Li, Shashi Kant Tiwari, Yue Qin, Edwin Yau, Ana Sanchez, Gatikrushna Singh, Kungyen Chang, Marcus Kaul, Maile Ann Young Karris, Tariq M. Rana

**Affiliations:** a Division of Genetics, Department of Pediatrics, UCSD Center for AIDS Research, and Institute for Genomic Medicine, University of California San Diego, La Jolla, California, USA; b Sanford Burnham Prebys Medical Discovery Institute, La Jolla, California, USA; c School of Medicine, Division of Biomedical Sciences, University of California, Riverside, California, USA; d Division of Infectious Diseases, UCSD Center for AIDS Research, Department of Medicine, University of California San Diego, La Jolla, California, USA; The Scripps Research Institute; Columbia University/HHMI

**Keywords:** long noncoding RNAs, epigenetic regulation, HIV promoter, ribonucleoprotein complexes, prevention of HIV-1 recrudescence

## Abstract

A major challenge in finding a cure for HIV-1/AIDS is the difficulty in identifying and eradicating persistent reservoirs of replication-competent provirus. Long noncoding RNAs (lncRNAs, >200 nucleotides) are increasingly recognized to play important roles in pathophysiology. Here, we report the first genome-wide expression analysis of lncRNAs in HIV-1-infected primary monocyte-derived macrophages (MDMs). We identified an lncRNA, which we named HIV-1-enhanced lncRNA (*HEAL*), that is upregulated by HIV-1 infection of MDMs, microglia, and T lymphocytes. Peripheral blood mononuclear cells of HIV-1-infected individuals show elevated levels of *HEAL*. Importantly, *HEAL* is a broad enhancer of multiple HIV-1 strains because depletion of *HEAL* inhibited X4, R5, and dual-tropic HIV replications and the inhibition was rescued by *HEAL* overexpression. *HEAL* forms a complex with the RNA-binding protein FUS, which facilitates HIV replication through at least two mechanisms: (i) *HEAL*-FUS complex binds the HIV promoter and enhances recruitment of the histone acetyltransferase p300, which positively regulates HIV transcription by increasing histone H3K27 acetylation and P-TEFb enrichment on the HIV promoter, and (ii) *HEAL*-FUS complex is enriched at the promoter of the cyclin-dependent kinase 2 gene, *CDK2*, to enhance CDK2 expression. Notably, *HEAL* knockdown and knockout mediated by RNA interference (RNAi) and CRISPR-Cas9, respectively, prevent HIV-1 recrudescence in T cells and microglia upon cessation of azidothymidine treatment *in vitro*. Our results suggest that silencing of *HEAL* or perturbation of the *HEAL*-FUS ribonucleoprotein complex could provide a new epigenetic silencing strategy to eradicate viral reservoirs and effect a cure for HIV-1/AIDS.

## Abstract (precis)

Despite our increased understanding of the functions of lncRNAs, their potential to develop HIV/AIDS cure strategies remains unexplored. A genome-wide analysis of lncRNAs in HIV-1-infected primary monocyte-derived macrophages (MDMs) was performed, and 1,145 differentially expressed lncRNAs were identified. An lncRNA named HIV-1-enhanced lncRNA (*HEAL*) is upregulated by HIV-1 infection and promotes HIV replication in T cells and macrophages. *HEAL* forms a complex with the RNA-binding protein FUS to enhance transcriptional coactivator p300 recruitment to the HIV promoter. Furthermore, *HEAL* knockdown and knockout prevent HIV-1 recrudescence in T cells and microglia upon cessation of azidothymidine treatment, suggesting *HEAL* as a potential therapeutic target to cure HIV-1/AIDS.

## INTRODUCTION

Human immunodeficiency virus type 1 (HIV-1) is a pathogenic retrovirus and the causative agent of AIDS and AIDS-related disorders. There were 1.7 million new infections globally in 2018, and ∼38 million people are currently living with HIV-1 (1). Although the introduction of antiretroviral therapy (ART) has prevented millions of AIDS-related deaths worldwide, patients must continue to receive ART for the remainder of their lives. HIV-1 reservoirs persist even while subjects are on ART, leading to a rapid increase in viral replication when therapy is discontinued (2). Therefore, eradication of persistent HIV-1 reservoirs remains the main barrier to achieving a cure for HIV-1/AIDS.

The prevailing view of persistence suggests that the virus remains in a latent state in memory CD4^+^ T cells regardless of plasma viral loads, allowing the virus to establish a lifelong infection in the host (3–5). Since the latent virus is refractory to existing antiretroviral therapies, curative strategies are now focusing on agents that reactivate viral replication and render it susceptible to conventional therapy. Any strategy aimed at controlling and eradicating viral reservoirs in HIV-1-infected individuals must target such latent reservoirs (6). In addition to CD4^+^ T cells, cells of the monocyte/macrophage lineage are well-established HIV-1 hosts (7–9). HIV-1-infected macrophages have been identified in the spinal cord, lymph nodes, and lung (10, 11). Because of the challenges in analyzing tissue macrophages, however, their contribution to viral replication and persistence has been difficult to assess.

The mammalian genome contains thousands of long noncoding RNAs (lncRNAs, >200 nucleotides), including intergenic lncRNAs (lincRNAs), which are increasingly recognized to play major roles in gene regulation (12). lncRNAs are transcribed in cells, but they lack protein-encoding potential (13, 14). It is estimated that the number of lncRNAs in humans ranges from 20,000 to over 100,000 (15, 16). The pathophysiological functions and mechanisms of lncRNAs in gene regulation have started to emerge (17, 18). lncRNA loci can regulate gene expression in a *cis* or *trans* manner, and these classifications provide a basic framework to design experimental approaches and understand lncRNA functions (19).

Work over the last few years has begun to uncover the role of lncRNAs in modulating HIV-1 gene expression (20–23; reviewed in reference 24). The first evidence that lncRNAs might be involved in HIV-1 replication came from experiments in the Jurkat T cell line, in which knockdown (KD) of *NEAT1* increased viral production by enhancing the nuclear export of Rev-dependent instability element (INS)-containing HIV-1 mRNAs (23). RNA interference (RNAi)-mediated silencing of an lncRNA, *NRON*, increased HIV-1 replication by stimulating NFAT (nuclear factor of activated T cells) and viral long terminal repeat (LTR) activities (21). In addition, *NRON* has been reported to suppress viral transcription by inducing Tat protein degradation, thus contributing to HIV-1 latency (25). An lncRNA, uc002yug.2, has been reported to activate latent HIV-1 replication through RUNX 1b/1c regulation and promoting Tat protein expression (20). Another lincRNA, MALAT1 (metastasis-associated lung adenocarcinoma transcript 1), promotes HIV transcription apparently by displacing the polycomb repressive complex 2 (PRC2) (26). Further, deep sequencing of HIV-1-infected CD4^+^ T cells has identified changes in a large number of lncRNAs (22), suggesting vital roles of lncRNAs in HIV-1 replication.

Here, we report the first genome-wide analysis of lncRNA expression in HIV-1-infected primary monocyte-derived macrophages (MDMs). We identified an lncRNA, which we named HIV-1-enhanced lncRNA (*HEAL*), that is conserved only in chimpanzees and rhesus monkeys, suggesting that it is a recently emerged gene. We found that *HEAL* regulates HIV-1 replication in microglia and T cells and does so by forming an RNA-protein complex with FUS RNA-binding protein, which is specifically enriched at the CDK2 promoter. *HEAL*-FUS complex positively regulates HIV transcription by recruitment of histone acetyltransferase p300 to the HIV promoter. Moreover, *HEAL* expression is elevated in peripheral blood mononuclear cells (PBMCs) from HIV-1-infected individuals. Remarkably, *HEAL* silencing by RNAi or knockout by CRISPR-Cas9 in T cells and microglia prevents recrudescence of HIV-1 upon withdrawal of azidothymidine (AZT) treatment *in vitro*. Thus, our results suggest that *HEAL* plays a vital role in HIV/AIDS pathogenesis and could potentially be exploited as a therapeutic target.

## RESULTS

### Genome-wide lncRNA expression analysis of HIV-1-infected primary monocyte-derived macrophages.

To identify lncRNAs involved in HIV replication, we designed a custom microarray. cDNA sequences of all known human lncRNAs were extracted from two sources and used for probe design: 1,703 defined lncRNA transcripts were from the Ensembl database and 2,915 transcripts were from the Havana database, as previously reported (27). Overall, 5 to 8 probes were designed per transcript, and ∼26,000 commercially available mRNA probes were also included in the array for quality control. To identify changes in lncRNA expression, MDMs from two healthy donors were infected with the macrophage-tropic HIV-1^BaL^ isolate, and RNA samples were prepared for microarray analysis after 3 days. We confirmed the HIV-1 infection efficiency of these cells by quantifying levels of *GP120* mRNA (see below) as well as mRNAs representative of the host antiviral immune response (interferon-induced guanylate-binding protein 1 [GBP1] and interferon-induced protein with tetratricopeptide repeats 1 [IFIT1]) (data not shown). MDMs from both donors showed a more vigorous response to HIV-1 infection at 3 days than at 6 days (data not shown); therefore, lncRNA expression was analyzed at 3 days postinfection.

**FIG 1:**
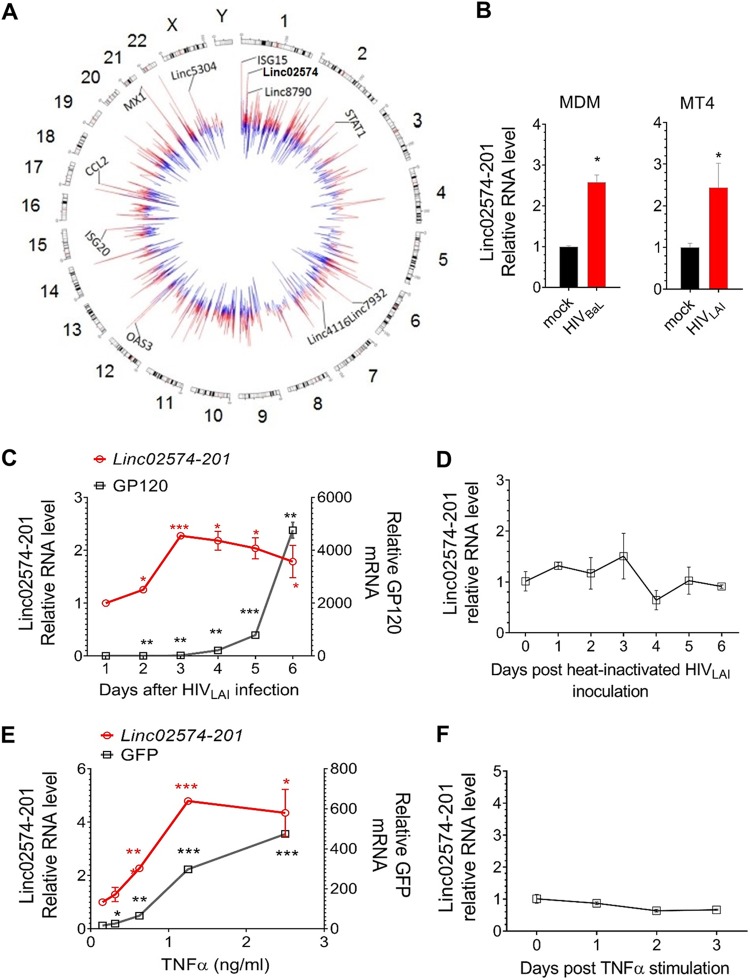
Identification of lncRNAs associated with HIV-1 replication. (A) Circos plot showing differentially expressed coding genes and lncRNAs in macrophages upon HIV-1 infection (*P* < 0.1). The length of each line is proportional to the log_2_ fold change in gene expression. The genes are represented according to their chromosomal locations. Upregulated and downregulated genes are shown in red and blue, respectively. As examples, six coding genes (ISG15, STAT1, OAS3, ISG20, CCL2, and MX1) and five noncoding genes (linc02574-201, linc8790, linc7932, linc4116, and linc5304) are indicated. (B) RT-qPCR analysis of linc02574-201 3 days after HIV infection of monocyte-derived macrophages (MDM) (left) or 2 days after infection of MT4 cells (right). Signals were normalized to *GAPDH* mRNA levels. *n* = 3, mean ± SD; *, *P < *0.05. (C and D) Kinetics of *GP120* mRNA and linc02574-201 expression in HIV-infected (C) or heat-inactivated HIV-inoculated (D) H9 cells. Signals were normalized to *GAPDH* mRNA levels. Results are the mean ± SD from three independent experiments. *, *P* < 0.05; **, *P* < 0.01; ***, *P* < 0.001. (E and F) RT-qPCR analysis of *GP120* mRNA and *HEAL* (linc02574-201) RNA expression in latently infected E4 cells 18 h after TNF-α treatment (E) or in Jurkat cells stimulated with TNF-α (3 ng/ml) for the indicated time points (F). Signals were normalized to *GAPDH* mRNA levels. Results are the mean ± SD from three independent experiments. *, *P* < 0.05; **, *P* < 0.01; ***, *P* < 0.001.

For analysis, lncRNAs were classified as HIV-1 modulated if their expression was suppressed or induced in MDMs from both donors by at least 1.5-fold with a *P* value of <0.05. We identified 1,145 unique lncRNAs (1,866 probes) that satisfied these criteria, of which 51% were suppressed and 49% were induced. A Circos plot was constructed to show the differentially expressed coding genes and lncRNAs (Fig. 1A; see also Table S1 in the supplemental material). The upregulated and downregulated genes are shown in red and blue, respectively, and the lengths of the colored lines indicate the log_2_ fold change in expression. As shown on the plot, the five lncRNAs with the highest fold change in expression upon HIV-1 infection were linc02574-201, linc8790, linc7932, linc4116, and linc5304. In addition, significant changes were observed in the expression of coding genes involved in the host response to infection (Fig. 1A) (e.g., ISG15, STAT1, OAS3, ISG20, CCL2, and MX1), confirming robust HIV-1 infection of these cells. We also analyzed the expression of linc02574-201, the most significantly induced lincRNA, using RT-qPCR and confirmed its upregulation upon HIV-1 infection of primary MDMs (Fig. 1B, left). To determine whether linc02574-201 was also upregulated in HIV-1-infected T cells, we examined two susceptible human CD4^+^ T cell lines, MT4 (28) and H9 (29). Both cell lines support infection with the T-cell-tropic HIV-1^LAI^ isolate, but viral replication peaks at a later time in H9 cells. RT-qPCR analysis showed that linc02574-201 was upregulated by HIV-1 infection of MT4 cells at 2 days postinfection (Fig. 1B). In H9 T cells, linc02574-201 expression was enhanced within 2 days of infection, reached a peak on day 3, and remained elevated for several days (Fig. 1C). To evaluate whether linc02574-201 regulation depends upon HIV replication, heat-inactivated HIV-1^LAI^ virus was inoculated in H9 cells and linc02574-201 was quantified at different time points. During the 6-day period of analysis, linc02574-201 was not changed by inactivated particles, indicating that HIV replication was essential for linc02574-201 upregulation in T cells (Fig. 1D).

10.1128/mBio.02016-19.5 TABLE S1 Gene expression changes in HIV-1-infected MDMs derived from two donors (donors 98 and 100) compared with uninfected MDMs. Related to Fig. 1. Download Table S1, XLSX file, 2.3 MB. Copyright © 2019 Chao et al. 2019 Chao et al. This content is distributed under the terms of the Creative Commons Attribution 4.0 International license.

### Expression of lncRNA *HEAL* correlates with HIV-1 replication.

To confirm the correlation between HIV-1 replication and linc02574-201 expression, we measured its levels in a latently HIV-1-infected Jurkat T cell line, E4, which carries a single integrated provirus and a short-lived variant of green fluorescent reporter protein (d2EGFP) in place of the *nef* gene (30). Treatment of E4 cells with tumor necrosis factor alpha (TNF-α) to reactivate HIV-1 replication not only induced an increase in EGFP fluorescence, as expected, but also increased linc02574-201 transcription (Fig. 1E), confirming the observations in H9 cells that this lincRNA is strongly associated with HIV-1 replication. To rule out the possibility that TNF-α alone can induce linc02574-201 expression, we stimulated Jurkat cells with 100 ng/ml TNF-α and analyzed lincRNA expression, and the results showed that TNF-α did not affect linc02574-201 expression levels (Fig. 1F). Therefore, we named linc02574-201 “*HEAL*” for HIV-1-enhanced lncRNA. Since *HEAL* was the most highly upregulated lincRNA examined and was induced by both HIV-1^BaL^ and HIV-1^LAI^ in primary MDMs and T cell lines, respectively, we further investigated its potential role in HIV-1 replication.

### lncRNA *HEAL* regulates HIV-1 replication.

**FIG 2:**
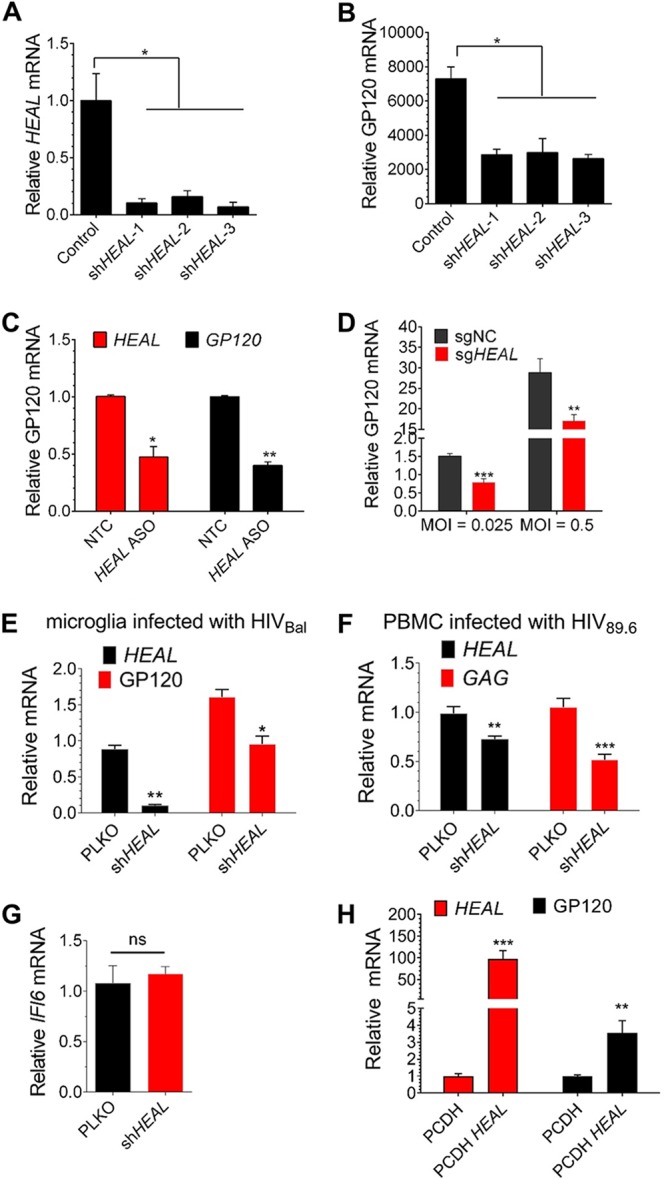
An HIV-enhanced lincRNA transcript (*HEAL*), linc02574-201, regulates HIV-1 replication. (A and B) Efficient silencing of *HEAL* in MT4 cells inhibits HIV-1 replication. MT4 cells expressing control vector or one of three shRNAs targeting *HEAL* were infected with HIV-1. *HEAL* (A) and *GP120* mRNA (B) were quantified by RT-qPCR at 2 days postinfection. Signals were normalized to *GAPDH* mRNA levels. *n* = 3, mean ± SD; *, *P* < 0.05. (C) Antisense oligonucleotides targeting *HEAL* inhibit HIV-1 replication. H9 cells were infected with lentiviruses encoding a nontargeting control (NTC) oligonucleotide or a *HEAL*-specific antisense oligonucleotide (*HEAL* ASO) for 2 days and then infected with HIV-1. The cells were reseeded for 3 days and then reinfected with NTC or ASO lentiviruses. Four days after the second ASO treatment, *GP120* mRNA levels were analyzed by RT-qPCR. Signals were normalized to *GAPDH* mRNA levels. *n* = 3, mean ± SD; *, *P* < 0.05; **, *P* < 0.01. (D) sgRNA targeting *HEAL* inhibits HIV-1 replication. MT4 cells were transduced with lentiviruses containing a nontargeting control sgRNA (sgNC) or a *HEAL*-specific sgRNA (sg*HEAL*) for 7 days and then infected with LAI at an MOI of 0.025 or 0.5. *GP120* mRNA levels were measured by RT-qPCR 2 days after infection. *n* = 3, mean ± SD; **, *P* < 0.01; ***, *P* < 0.001. (E) Knockdown of *HEAL* inhibits HIV^BaL^ replication. Microglial cells expressing control vector or sh*HEAL*-1 were infected with HIV^BaL^. *HEAL* and *GP120* mRNAs were quantified by RT-qPCR at 9 days postinfection. Results are the mean ± SD from three independent experiments. Signals were normalized to *GAPDH* mRNA levels. *, *P* < 0.05; **, *P* < 0.01. (F) Knockdown of *HEAL* inhibits HIV^89.6^ replication in primary PBMCs. Activated primary PBMCs were transduced with control or sh*HEAL*-1 lentivirus for 7 days. After activating for the second time for 3 days, cells were infected with HIV^89.6^ at an MOI of 0.01. *HEAL* and *GP120* mRNA were quantified by RT-qPCR at 3 days postinfection. Signals were normalized to *GAPDH* mRNA levels. *n* = 3, mean ± SD; **, *P* < 0.01; ***, *P* < 0.001. (G) *HEAL* knockdown did not affect *IFI6* expression. *IFI6* mRNA expression was quantified in MT4 cells expressing control vector or shRNA targeting *HEAL*. ns, not significant. (H) Overexpression of *HEAL* enhances HIV-1 replication. *HEAL* was overexpressed in MT4 cells using a pCDH lentiviral vector, and the cells were infected with HIV-1 2 days later. *HEAL* and *GP120* mRNA levels were measured by RT-qPCR 2 days after infection. Signals were normalized to *GAPDH* mRNA levels. *n* = 3, mean ± SD; **, *P* < 0.01; ***, *P* < 0.001.

To determine the functional significance of *HEAL* induction by HIV-1, we examined viral replication in T cells in which *HEAL* expression was silenced by three short hairpin RNAs (shRNAs) targeting different regions of *HEAL*. MT4 T cells were transduced with control (pLKO empty vector) or shRNA-carrying lentiviruses for 2 days and then infected with HIV-1^LAI^ for an additional 2 days, at which time *HEAL* RNA and HIV-1 *GP120* mRNA were quantified by RT-qPCR. All three shRNAs not only effectively silenced *HEAL* expression (Fig. 2A) but also strongly reduced *GP120* mRNA levels (Fig. 2B). To confirm this using an alternative approach, H9 cells were transfected with an antisense oligonucleotide (ASO) targeting *HEAL*. Here too, *HEAL* silencing reduced viral replication, as reflected by *GP120* mRNA levels, compared with cells transfected with a control ASO (Fig. 2C). We next asked whether editing the genomic sequence of *HEAL* would inhibit HIV replication. MT4 cells were transduced with Cas9 and single guide RNA (sgRNA) specific to *HEAL* exon2. Similar to other strategies in Fig. 2B and C, editing *HEAL* also decreased HIV replication (Fig. 2D). To investigate if *HEAL* could be a universal HIV enhancer, we knocked down *HEAL* in a microglia cell line and infected it with HIV^BaL^, an R5-tropic strain, and the results showed that replication of R5-tropic virus was decreased by *HEAL* silencing (Fig. 2E). Additionally, replication of a dual-tropic virus (HIV^89.6^) was dependent on *HEAL* expression (Fig. 2F). These results demonstrate that *HEAL* is a broad enhancer of HIV replication. The finding that *HEAL* expression regulates HIV-1 prompted us to examine its expression in host tissues with prominent roles in immunity. Interestingly, *HEAL* was expressed in a very narrow range of tissues, with high expression being detected only in adrenal glands, thymus, and skeletal muscle (Fig. S1A). Since the thymus is a specialized primary lymphoid organ, this result provided further support for a link between *HEAL* and HIV-1 replication.

10.1128/mBio.02016-19.1 FIG S1 Related to Fig. 2. HEAL expression correlates with HIV replication. (A) RT-qPCR analysis of *HEAL* expression in human tissues. Results are the mean ± SD from triplicate wells and are normalized to *GAPDH.* (B) Mapping of the *HEAL* sequence by rapid amplification of cDNA ends (RACE). Products of 3′ and 5′ RACE are shown at ∼500 bp. (C) Reexpression of *HEAL* in *HEAL* knockdown cells increases HIV-1 replication. RT-qPCR of *HEAL* RNA and *GP120* mRNA in MT4 cells. Cells were infected with control (pLKO empty vector), *HEAL* overexpression, or *HEAL* shRNA lentiviruses and then infected with HIV-1 3 days later. Results are the mean ± SD from three independent experiments. Signals were normalized to *GAPDH* mRNA levels. *, *P* < 0.05; **, *P* < 0.01; ***, *P* < 0.001; ****, *P* < 0.0001. (D) *HEAL* is a recently emerged gene. Comparative genomic alignment of seven species to the human genome at the 5′ and 3′ ends of *HEAL* (RP11-288L9.1; top, in blue). Chromosome numbers are indicated by the color key shown below. Download FIG S1, PDF file, 0.2 MB. Copyright © 2019 Chao et al. 2019 Chao et al. This content is distributed under the terms of the Creative Commons Attribution 4.0 International license.

Having established that *HEAL* silencing suppresses HIV-1 replication, we asked if the inverse is true: can *HEAL* overexpression enhance HIV-1 replication? We first mapped the full-length sequence of *HEAL* by performing 5′ and 3′ rapid amplification of cDNA ends (RACE) (31, 32). We identified *HEAL* as a 467-bp transcript of gene RP11-288L9 located on human chromosome 1 (Fig. S1B). Only one transcript, the reverse strand downstream of gene *IFI6*, was identified. However, KD of *HEAL* had no significant effect on *IFI6* expression (Fig. 2G), indicating that *IFI6* was not the functional target of *HEAL*. Next, we examined the effects of lentivirus-mediated overexpression of *HEAL* on HIV-1 infection in MT4 cells by examining GP120 expression 2 days after HIV-1 infection. We found that *HEAL* overexpression upregulated HIV-1 replication compared with control cells (Fig. 2H). Consistent with this, rescue experiments showed that *HEAL* reexpression in *HEAL* KD cells increased *GP120* mRNA levels (Fig. S1C). Taken together, the *HEAL* KD, overexpression, and rescue experiments demonstrate an important functional role for *HEAL* in HIV-1 replication. We performed a comparative genomic analysis of the *HEAL* sequence in different species to determine whether *HEAL* is evolutionarily conserved. Intriguingly, *HEAL* was highly conserved in only chimpanzees and rhesus monkeys, suggesting that this lincRNA could be a recently emerged gene that is exploited by HIV-1 (Fig. S1D). It is tempting to speculate that the narrow species expression of *HEAL* might play an important role in host specificity for HIV-1 replication.

### *HEAL* directly binds to the HIV-1 promoter.

**FIG 3:**
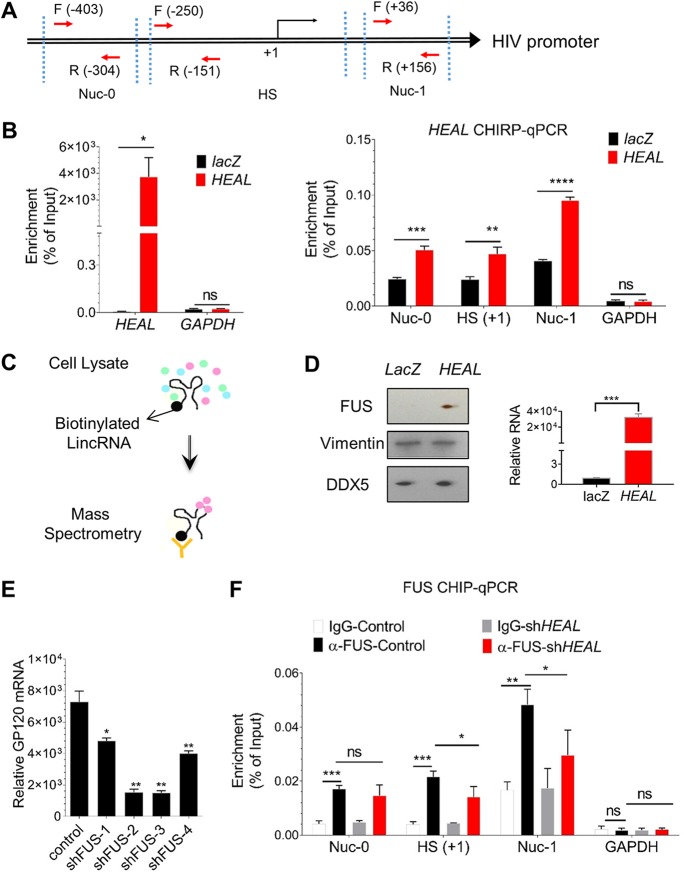
*HEAL* forms a complex with FUS protein and binds to HIV promoter. (A) Schematic of HIV promoter regions based on nucleosome architecture. Primers to identify different regions of the promoter are indicated. Nuc-0, nucleosome 0 region. HS, DNase I highly sensitive region. Nuc-1, nucleosome 1 region. (B) *HEAL* is recruited to the HIV promoter. ChIRP assays were performed in HIV-infected MT4 cells using a nonspecific *lacZ* probe or *HEAL*-specific probes. Specificity of *HEAL* probes (left) and enrichment at the HIV promoter (right) are shown. *GAPDH* mRNA (left) or genomic GAPDH is shown as negative control. Probes are listed in Table S4. Mean ± SD of *n* = 3. *, *P* < 0.05; **, *P* < 0.01; ***, *P* < 0.001; ****, *P* < 0.0001; ns, not significant. (C) Experimental design for purification and identification of *HEAL*-associated cellular proteins using biotinylated *HEAL* or *lacZ* (control) RNA pulldown followed by mass spectrometry. (D) Immunoblotting of FUS, vimentin, and DDX5 proteins identified from biotinylated *lacZ* and *HEAL* pulldown assays. Vimentin and DDX5 are shown as negative controls. *HEAL* mRNA enrichment in pulldown fraction was detected using qPCR. Mean ± SD of *n* = 3. ***, *P* < 0.001. (E) *FUS* knockdown inhibits HIV-1 replication. MT4 cells were infected with control lentivirus (empty vector, pLKO) or lentiviruses carrying *FUS*-targeting shRNAs. Two days later, they were infected with HIV-1, and *GP120* mRNA was quantified by RT-qPCR analysis after 2 days. Signals were normalized to *GAPDH* mRNA levels. *n* = 3, mean ± SD; *, *P* < 0.05; **, *P* < 0.01. (F) FUS recruitment to the HIV promoter is dependent on *HEAL*. HIV-infected control or *HEAL* knockdown MT4 cells were prepared for FUS-CHIP analysis. RT-qPCR of the HIV promoter regions or GAPDH region coimmunoprecipitated with FUS was performed. Mean ± SD of *n* = 3. *, *P* < 0.05; **, *P* < 0.01; ***, *P* < 0.001.

To investigate the mechanism by which *HEAL* regulates HIV replication, chromatin isolation by RNA purification (ChIRP) assays (32–34) were performed to assess *HEAL* binding to the HIV promoter. Chromatin fractions from HIV-infected MT4 cells were incubated with biotinylated *HEAL* or control partial *lacZ* (without protein-encoding potential) RNA and analyzed by RT-qPCR for the presence of HIV promoter sequences. Three PCR primers (Nuc-0, HS, and Nuc-1) spanning from −403 to +156 relative to the +1 transcription start site were designed based on the nucleosome structure of the HIV-1 5′ long terminal repeat (LTR) region (Fig. 3A). We observed that biotinylated *HEAL*, but not *GAPDH*, mRNA was significantly enriched compared to the *lacZ* probe (Fig. 3B, left), showing that specific *HEAL* ChIRP was successful. Genomic glyceraldehyde-3-phosphate dehydrogenase (GAPDH) is shown as a negative control (Fig. 3B, right). Importantly, RT-qPCR analysis of the sequences pulled down with *HEAL* showed specific enrichment of the promoter regions (Fig. 3B). These results indicate that *HEAL* directly binds to the HIV promoter in order to regulate viral replication.

10.1128/mBio.02016-19.8 TABLE S4 Oligonucleotides used in this study. Related to Materials and Methods. Download Table S4, DOCX file, 0.02 MB. Copyright © 2019 Chao et al. 2019 Chao et al. This content is distributed under the terms of the Creative Commons Attribution 4.0 International license.

### The RNA-binding protein FUS interacts with *HEAL* to regulate HIV replication.

Many lncRNAs have been shown to regulate gene expression by interacting with RNA-binding proteins, transcription factors, or chromatin-modifying complexes (31, 32, 34, 35). We hypothesized that *HEAL* regulates HIV promoter activity by interacting with RNA-binding proteins. To test this, we used an unbiased approach (Fig. 3C) in which biotinylated *HEAL* or *lacZ* was incubated with MT4 cell lysates, and proteins associated with the biotinylated RNAs were pulled down with streptavidin-conjugated beads, eluted, and analyzed by mass spectrometry. The RNA-binding protein FUS (fused in sarcoma) was identified as the most likely *HEAL* cofactor based on the number of enriched peptides present in *HEAL* versus *lacZ* samples (Table S5). We verified that FUS specifically interacts with *HEAL* by performing Western blot analysis of *HEAL*- and *lacZ*-associated proteins. Indeed, FUS was present specifically in the *HEAL* pulldown samples, whereas the intermediate filament protein vimentin and the RNA-binding protein DDX5, probed as controls, were enriched in both *HEAL* and *lacZ* samples (Fig. 3D). These results confirmed that *HEAL* and FUS can form a ribonucleoprotein complex *in vivo*.

10.1128/mBio.02016-19.9 TABLE S5 Peptides enriched in *HEAL*-precipitated fraction. Related to Fig. 3C. Download Table S5, XLS file, 0.4 MB. Copyright © 2019 Chao et al. 2019 Chao et al. This content is distributed under the terms of the Creative Commons Attribution 4.0 International license.

We next asked whether FUS could modulate HIV-1 replication. FUS expression was silenced in MT4 cells using four different shRNAs, and the cells were then infected with HIV-1. Knockdown of FUS dramatically decreased the levels of *GP120* mRNA (Fig. 3E), suggesting that FUS protein binds to *HEAL* to coregulate HIV transcription. To test this, we performed chromatin immunoprecipitation (ChIP) analyses in HIV-1-infected control and *HEAL* knockdown MT4 cells. After immunoprecipitation of endogenous FUS protein, the associated DNA was eluted and examined by RT-qPCR for the presence of HIV promoter sequences. This analysis confirmed that FUS binds to the HIV promoter in HIV-infected cells (Fig. 3F). Genomic GAPDH was not enriched and is shown as a negative control (Fig. 3F, right). Importantly, compared to control cells, FUS binding on HS and Nuc-1 regions was significantly decreased by *FUS* knockdown (Fig. 3F), further supporting the existence of a *HEAL*-FUS-HIV regulator*y* axis that controls HIV-1 replication.

### *HEAL-*FUS complex recruits p300 to increase the H3K27ac modification and P-TEFb loading on the HIV promoter.

**FIG 4:**
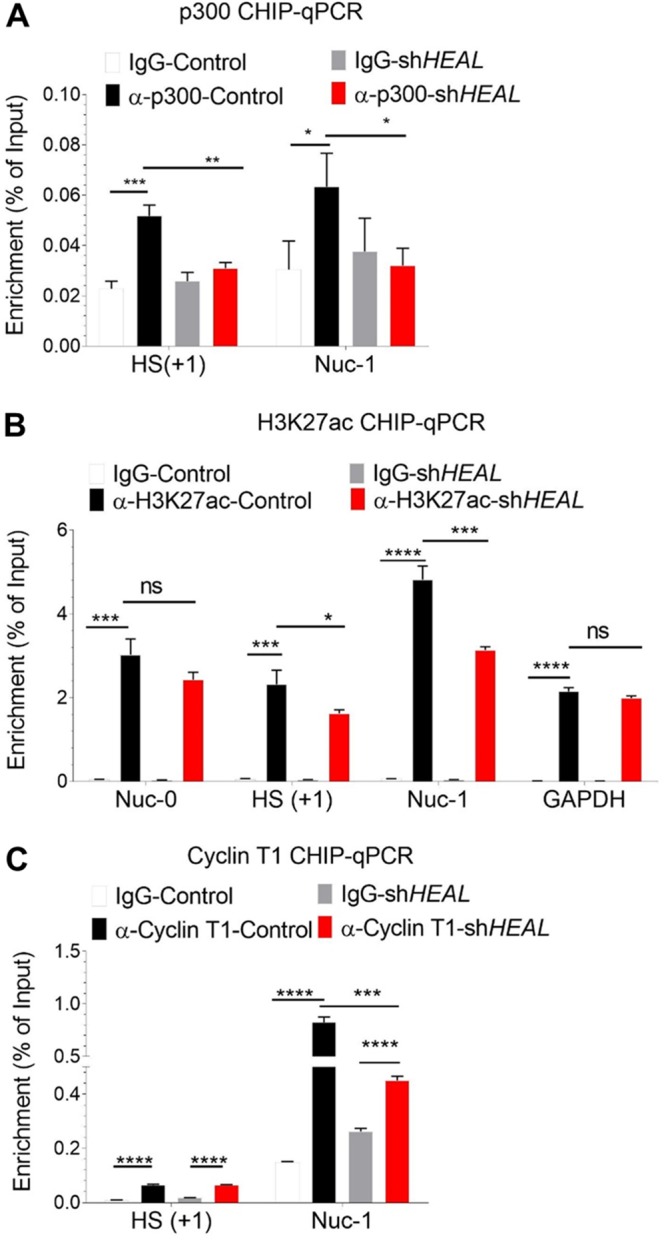
*HEAL*-FUS complex recruits histone acetyltransferase p300 to modulate histone modification and P-TEFb enrichment at the HIV promoter. HIV-infected control or *HEAL* knockdown MT4 cells were prepared for CHIP analyses—p300 (A), H3K27ac (B), and cyclin T1 (C)—as described in the legend to Fig. 3F. Results of RT-qPCR of the HIV promoter regions enriched for p300, H3K27ac, and cyclin T1 are shown in panels A, B, and C, respectively. Mean ± SD of *n* = 3. *, *P* < 0.05; **, *P* < 0.01; ***, *P* < 0.001; ****, *P* < 0.0001; ns, not significant.

During active HIV transcription in cells, histone acetyltransferase (HAT) complex is recruited to the HIV promoter region that acetylates histone residues, leading to enhanced transcription (36–38). FUS has been shown to interact with HAT complex members, including p300, CBP, and TIP60 (39). Based on the findings that FUS-*HEAL* complex binds to the HS and Nuc-1 regions of the HIV promoter (Fig. 3F), we hypothesized that FUS-*HEAL* complex might enhance HAT complex recruitment to the HIV promoter. To test this hypothesis, we performed p300-CHIP and H3K27ac-CHIP in control and *HEAL* knockdown T cells infected with HIV. Our results showed that p300 recruitment and H3K27ac modification were significantly decreased in *HEAL*-depleted cells, especially in HS and Nuc-1 regions, which correspond to *HEAL*-FUS complex binding regions (Fig. 3F and Fig. 4A and B). Control experiments showed that H3K27ac modification in the GAPDH genomic region was unchanged by HEAL knockdown (Fig. 4B). These results suggested that *HEAL*-FUS complex facilitated the binding of p300 acetyltransferase to the HIV promoter, thus enhancing H3K27ac modification and HIV transcription. During HIV transcription, a positive transcription elongation factor, P-TEFb, binds HIV Tat-TAR RNA complex to relieve elongation blocks by phosphorylating RNA polymerase (Pol) II and negative elongation factors such as SPT5 and SDIF (37, 38, 40, 41). To further confirm whether HIV transcription was enhanced by *HEAL*, we analyzed the binding of a P-TEFb subunit, cyclin T1, on the HIV promoter. Our CHIP-qPCR experiments showed that cyclin T1 was significantly enriched on the HS and Nuc-1 regions of the HIV promoter, with a higher binding in the Nuc-1 region than the HS region as predicted from the elongation function of P-TEFb (Fig. 4C). Importantly, cyclin T1 binding was significantly reduced in *HEAL* KD cells (Fig. 4C). Altogether, these results demonstrate that *HEAL* plays an important role in enhancing p300 binding to the HIV promoter and positively regulating HIV transcription.

### *HEAL* stimulates CDK2 expression.

**FIG 5:**
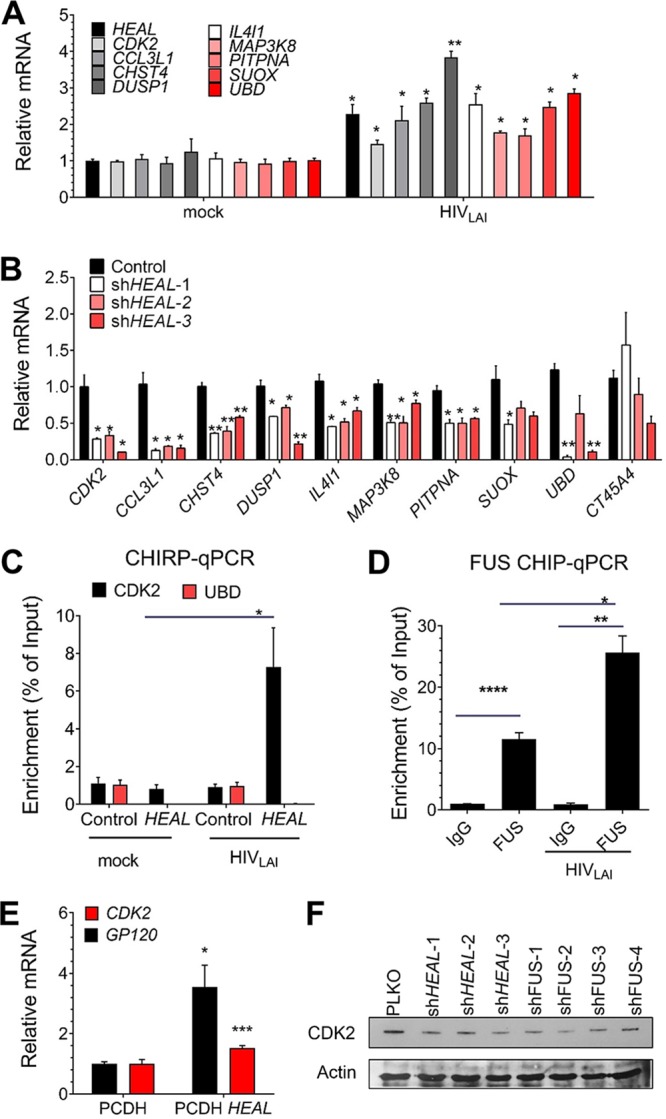
*HEAL* is required to maintain expression of CDK2 to support HIV-1 replication. (A) RT-qPCR analysis of *HEAL*-regulated mRNAs in uninfected or HIV-1-infected MT4 cells 2 days postinfection. Signals were normalized to *GAPDH* mRNA levels. *n* = 3, mean ± SD; *, *P* < 0.05; **, *P* < 0.01. (B) RT-qPCR analysis of *HEAL*-regulated mRNAs in MT4 cells expressing control vector or three *HEAL* shRNAs and then infected with HIV-1. mRNAs were quantified 2 days later. Signals were normalized to *GAPDH* mRNA levels. *n* = 3, mean ± SD; *, *P* < 0.05; **, *P* < 0.01. (C) qPCR of *HEAL*-associated DNA in ChIRP assays of uninfected or HIV-1-infected MT4 cells expressing empty vector (control) or overexpressing *HEAL*. *n* = 3, mean ± SD; *, *P* < 0.05. (D) FUS protein interaction with the *CDK2* promoter is enhanced by HIV-1 infection. ChIP assays of H9 T cells 7 days after infection with HIV-1. RT-qPCR analysis of the *CDK2* promoter region was performed on control IgG or anti-FUS immunoprecipitates. Signals were normalized to *GAPDH* mRNA levels. *n* = 3, mean ± SD; *, *P* < 0.05; **, *P* < 0.01; ****, *P* < 0.0001. (E) RT-qPCR analysis of *GP120* and CDK2 mRNA in HIV-1-infected MT4 cells expressing empty vector (pCDH) or overexpressing *HEAL*. Signals were normalized to *GAPDH* mRNA levels. *n* = 3, mean ± SD; *, *P* < 0.05; ***, *P* < 0.001. (F) Knockdown of *HEAL* or *FUS* reduces cellular CDK2 protein levels. MT4 cells were transduced with lentiviruses carrying empty vector or the indicated shRNAs and infected with HIV-1 2 days later. Cell lysates were prepared 2 days after infection and analyzed by Western blotting with anti-CDK2 or anti-β-actin antibodies.

We next sought to shed light on the mechanism by which *HEAL* might regulate HIV-1 replication by identifying host genes whose expression is controlled by *HEAL*. MT4 cells were transduced with *HEAL-*targeting or control shRNAs for 2 days and then infected with HIV-1. Genome-wide mRNA analysis was performed 2 days postinfection using a human HT-12 v4 expression BeadChip kit, which contains >47,000 probes derived from the NCBI RefSeq release 38, among other sources. Candidate *HEAL*-modulated genes were selected based on (i) the fold change (*P* < 0.05) in their expression in HIV-1-infected MT4 cells expressing control versus *HEAL-*specific shRNA and (ii) the number of detected probe*s*. Fifty percent of genes of the top hit were identified in both of the two *HEAL* KD cells. Fifteen genes showed decreased expression in *HEAL* KD HIV-1-infected cells, suggesting that they may be regulated by *HEAL*. Of these 15 genes, 9 were further validated by RT-qPCR analysis and shown to be upregulated in HIV-1-infected MT4 cells (Fig. 5A and Tables S2 and S3) and reduced by *HEAL* KD (Fig. 5B), consistent with the expected behavior of *HEAL*-regulated genes. CT45A4, which was not affected by HIV-1 infection in our genome-wide analysis, was used as a control for these experiments.

10.1128/mBio.02016-19.6 TABLE S2 Gene expression changes in *HEAL* knockdown (sh*HEAL*-1-expressing) and HIV-1-infected MT4 cells compared with uninfected cells. Related to Fig. 5. Download Table S2, XLSX file, 0.6 MB. Copyright © 2019 Chao et al. 2019 Chao et al. This content is distributed under the terms of the Creative Commons Attribution 4.0 International license.

10.1128/mBio.02016-19.7 TABLE S3 Gene expression changes in *HEAL* knockdown (sh*HEAL*-2-expressing) and HIV-1-infected MT4 cells compared with uninfected cells. Related to Fig. 5. Download Table S3, XLSX file, 0.5 MB. Copyright © 2019 Chao et al. 2019 Chao et al. This content is distributed under the terms of the Creative Commons Attribution 4.0 International license.

To determine whether *HEAL* regulates gene expression by direct interaction with promoters, we performed ChIRP assays. For this, biotinylated *HEAL* or control (*lacZ*) RNA was incubated with the chromatin fraction of uninfected or HIV-1-infected MT4 T cells, and the RNA was then pulled down with streptavidin-conjugated beads (Fig. S2A). DNA coprecipitated with *HEAL* or *lacZ* RNA was recovered and analyzed by qPCR. As shown in Fig. 5C, the *CDK2* promoter sequence was specifically pulled down by *HEAL* in HIV-1-infected cells but not in uninfected cells, identifying *CDK2* as a bona fide *HEAL* target. To investigate whether *HEAL*-FUS complex plays a role in CDK2 regulation, we performed a FUS-ChIP assay in mock- and HIV-infected cells. As shown in Fig. 5D, FUS could significantly bind the CDK2 promoter and was enhanced in HIV infection. These results confirmed *HEAL*-FUS complex as regulating CDK2 expression. Additionally, *HEAL* overexpression increased *CDK2* mRNA levels (Fig. 5E) while *HEAL* and FUS KD decreased CDK2 protein levels (Fig. 5F) in HIV-1-infected MT4 cells. Since the CDK2 activation requires its interaction with cyclin A (42), we investigated whether the decrease in CDK2 expression affects the formation of functional complexes of CDK2. We performed cyclin A immunoprecipitation to analyze the CDK2-cyclin A interactions in nontargeting shRNA control (NC) and *HEAL* KD cells. Our results showed that CDK2-cyclin interactions were not affected by *HEAL* KD, suggesting that CDK2 was functional in these KD cells (Fig. S2B). Collectively, these results demonstrate that *HEAL*-FUS complex binds to the promoter of CDK2 and regulates its expression.

10.1128/mBio.02016-19.2 FIG S2 Related to Fig. 3. ChIRP assays of HIV-1-infected T cells. (A) Cross-linked chromatin was incubated with biotinylated *HEAL* followed by streptavidin-conjugated beads. RNA pulled down by the beads was analyzed by RT-qPCR. *n* = 3, mean ± SD. *, *P* < 0.05; **, *P* < 0.01. (B) CDK2 binds to cyclin A. Western blot analysis of CDK2 and cyclin A in control mouse IgG or anti-cyclin A antibody immunoprecipitates from NC (control) or *HEAL* knockdown MT4 cells. Download FIG S2, PDF file, 0.1 MB. Copyright © 2019 Chao et al. 2019 Chao et al. This content is distributed under the terms of the Creative Commons Attribution 4.0 International license.

### *HEAL* silencing prevents reactivation of HIV-1 replication in T cells and microglial cells after cessation of AZT treatment.

**FIG 6:**
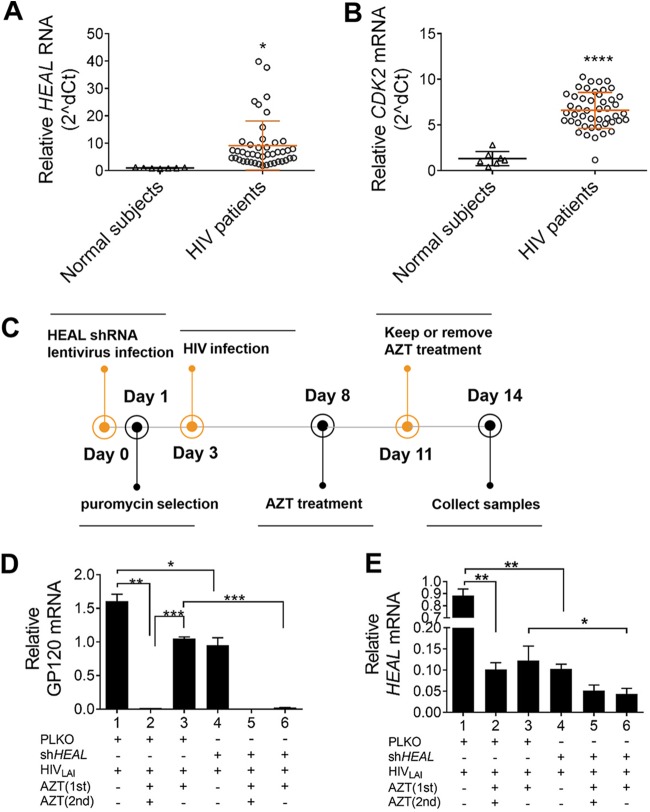
*HEAL* silencing maintains HIV-1 suppression after AZT withdrawal. (A and B) *HEAL* (A) and CDK2 (B) expression levels were increased in PBMCs of HIV-1-infected individuals. *HEAL* (A) and CDK2 (B) expression levels were measured by RT-qPCR, and signals were normalized to *GAPDH* mRNA levels. Data were calculated using the threshold cycle (2^−ΔΔ^*^CT^*) method and analyzed with Student’s *t* test. *, *P* < 0.05; ****, *P* < 0.0001. (C to E) Experimental design for the analysis of HIV-1 replication in *HEAL*-depleted and/or AZT-treated cells. H9 T cells were infected with control or *HEAL* shRNA-carrying lentiviruses, maintained under puromycin selection conditions for 3 days, and then infected with HIV-1. Cells were treated with AZT (0 to 20 μM) between days 8 and 11 and then either switched to AZT-free medium or maintained in medium containing 0 to 20 μM AZT for an additional 3 days (C). On day 14, cells were collected and analyzed for *GP120* mRNA (D) or *HEAL* RNA (E) by RT-qPCR. Signals were normalized to *GAPDH* mRNA levels. *n* = 3, mean ± SD; *, *P* < 0.05; **, *P* < 0.01; ***, *P* < 0.001; ns, not significant.

To determine whether *HEAL* expression is related to the level of HIV-1 infection *in vivo*, we compared its expression in PBMCs from 48 viably stored blood samples collected from 33 HIV-1-infected individuals. RT-qPCR analysis of PBMCs showed that both *HEAL* and *CDK2* mRNA expression was upregulated by HIV-1 infection, consistent with the findings in H9 and MT4 T cell lines (Fig. 6A and B).

Our finding that shRNA-mediated suppression of *HEAL* concomitantly inhibited HIV-1 replication in T cells suggests that *HEAL* silencing may effect a functional cure. We designed a strategy to determine the relationship between *HEAL* expression and viral rebound, a well-established clinical consequence of withdrawal from AZT therapy. H9 cells were infected with control or *HEAL* shRNA-carrying lentiviruses for 3 days and then infected with HIV-1 for 5 days. The cells were then treated with AZT for 3 days (first treatment), washed, and placed back in culture in AZT-free or AZT-containing medium (second treatment) for a further 3 days. *HEAL* RNA and *GP120* mRNA levels were quantified on days 11 and 14, after the first and second AZT/control treatments (Fig. 6C). We found that HIV-1 replication was effectively suppressed in cells expressing the shRNA control vector (pLKO) and treated with AZT for the entire 6 days (i.e., first and second treatments). However, removing AZT after the first treatment led to a dramatic rebound in HIV-1 replication, consistent with clinical observations (bars 1 to 3, Fig. 6D). Remarkably, HIV-1 replication remained suppressed in *HEAL* KD cells when AZT was removed (bars 5 and 6, Fig. 6D). In accord with results shown in Fig. 1 and 2, *HEAL* RNA expression correlated with HIV replication (Fig. 6D and E). This effect was confirmed in human microglial cells, where *GP120* expression could not be rescued by removal of AZT in *HEAL* KD cells (Fig. S3). Taken together, these results suggest that *HEAL* silencing might be exploited therapeutically to prevent HIV-1 rebound replication when ART is discontinued.

10.1128/mBio.02016-19.3 FIG S3 Related to Fig. 5. HEAL silencing maintains HIV-1 suppression after AZT withdrawal. (A) Experimental design for the analysis of HIV replication in human microglial cells. Cells were infected with control or *HEAL* shRNA lentiviruses, maintained under puromycin selection conditions for 8 days, and then infected with HIV-1. Cells were treated with AZT between days 12 and 15 and then switched to AZT-free medium or maintained in AZT for an additional 2 days. (B) Cells were analyzed for *GP120* mRNA expression on day 17. Signals were normalized to β-actin mRNA levels. *n* = 3, mean ± SD; **, *P* < 0.01; ***, *P* < 0.001; ****, *P* < 0.0001; ns, not significant. Download FIG S3, PDF file, 0.1 MB. Copyright © 2019 Chao et al. 2019 Chao et al. This content is distributed under the terms of the Creative Commons Attribution 4.0 International license.

### *HEAL* deletion by CRISPR prevents rebound of HIV-1 replication upon ART withdrawal.

**FIG 7:**
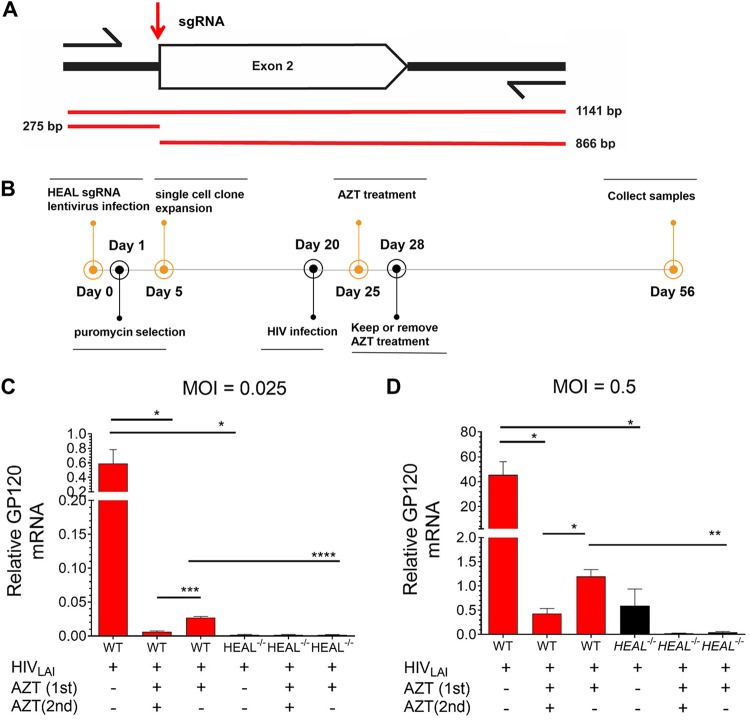
*HEAL* knockout maintains HIV-1 suppression after AZT withdrawal. (A) Location of the guide RNA used for CRISPR-Cas9-mediated editing of the *HEAL* locus in H9 cells. Arrows indicate the primers used to amplify the genomic region harboring the editing site. Red lines indicate the segments amplified and the segments predicted to be present in positive clones after T7EI digestion. (B) Experimental design for the analysis of HIV-1 replication in *HEAL*^−/−^ or control cells. H9 T cells were infected with control or *HEAL* sgRNA-carrying lentiviruses, maintained under puromycin selection conditions for 4 days, and then infected with HIV-1 at an MOI of 0.025 or 0.5. Cells were treated with AZT (20 μM) between days 25 and 28 and then switched to AZT-free or 20 μM AZT-containing medium for an additional 28 days. On day 56, cells were collected and analyzed. (C and D) RT-qPCR analysis of *GP120* mRNA in H9 cells infected at an MOI of 0.025 (C) or 0.5 (D). Signals were normalized to *GAPDH* mRNA levels. *n* = 3, mean ± SD; *, *P* < 0.05; **, *P* < 0.01; ***, *P* < 0.001; ****, *P* < 0.0001.

To further confirm the *HEAL* RNAi results (Fig. 6C and D) and determine the therapeutic potential of CRISPR-Cas9 to generate *HEAL* KO cells, we deleted *HEAL* in H9 cells by CRISPR-Cas9-mediated editing using an sgRNA targeting *HEAL* exon 2 (Fig. 7A). *HEAL* knockouts and control cells were obtained through single-cell clonal expansion of H9 cells transfected with *HEAL* or control sgRNA. Positive clones were identified by T7 endonuclease I (T7EI) assay, which generated two editing segments (275 bp and 866 bp) (Fig. S4A). We also amplified and sequenced the genomic region around the editing site and confirmed biallelic modification of the *HEAL* locus (Fig. S4B).

10.1128/mBio.02016-19.4 FIG S4 Related to Fig. 6. Identification of *HEAL* knockout cells. (A) *HEAL* knockout H9 clones were screened using T7EI analysis. WT, wild-type H9 cells. (B) Positive knockout clone 3 was sequenced to confirm biallelic modification of the *HEAL* locus. (C) *HEAL* knockout maintains HIV-1 suppression after AZT removal. HIV-1 rebound replication experiments were performed as described for Fig. 5. Signals were normalized to *GAPDH* mRNA levels. *n* = 3, mean ± SD; *, *P* < 0.05; **, *P* < 0.01. Download FIG S4, PDF file, 0.1 MB. Copyright © 2019 Chao et al. 2019 Chao et al. This content is distributed under the terms of the Creative Commons Attribution 4.0 International license.

Next, we performed HIV-1 rebound replication assays similar to the one described for Fig. 6C. We found that HIV-1 replication was effectively suppressed (∼80-fold) in *HEAL*^−/−^ cells compared with control cells and remained suppressed on days 11 and 14 after AZT removal (Fig. S4C). To test the long-term effect of *HEAL* knockout on suppression of HIV-1 rebound replication, we examined cultures 28 days after AZT withdrawal. *HEAL*^−/−^ H9 cells or control cells were infected with HIV-1 at multiplicities of infection (MOIs) of 0.025 or 0.5 for 5 days, treated with AZT for 3 days (first treatment), washed, and then cultured in AZT-free or AZT-containing medium for a further 28 days (second treatment; Fig. 7B). HIV-1 replication was effectively suppressed in *HEAL*^−/−^ cells, with ∼280-fold and ∼40-fold inhibition in cells infected at MOIs of 0.025 and 0.5, respectively. Remarkably, HIV-1 replication remained suppressed in *HEAL*^−/−^ cells, even when AZT had been removed 28 days earlier (Fig. 7C and D). Collectively, these results suggest that inhibition of *HEAL* expression could be a potential therapy to prevent HIV-1 replication.

## DISCUSSION

In this study, we systematically analyzed changes in the expression of lncRNAs upon HIV-1 infection of macrophages and MT4 and H9 T cells. Through a combination of genomic, biochemical, and cell biological approaches, we identified the novel lincRNA *HEAL* as a key player in controlling HIV-1 replication in macrophages, microglia, and T cells. We first found that HIV-1 infection markedly upregulated the expression of several lincRNAs in the T cell lines, of which *HEAL*, linc8790, linc7932, linc4116, and linc5304 were the most increased in MT4 cells, whereas *HEAL*, linc4116, and linc5304 were most highly increased in H9 cells (data not shown). These minor differences in upregulation suggest that the effect of HIV-1 on lncRNA expression may vary between different cells. The demonstration that *HEAL* expression is tissue and species specific supports the notion that it is a recently emerged gene and may contribute to the species restriction of HIV-1 replication (see Fig. S1A and D in the supplemental material).

*HEAL* expression was significantly upregulated in H9 at 2 days postinfection (Fig. 1C), while heat-inactivated virus did not change *HEAL* expression (Fig. 1D). These results suggest that the upregulation is not dependent on viral entry. Furthermore, in the cellular model of latent HIV infection, *HEAL* is induced upon HIV activation (Fig. 1E). These results confirm that *HEAL* regulation is dependent on HIV replication. Further studies are needed to define the precise triggers and signaling mechanism by which *HEAL* expression is induced after HIV-1 infection.

To our knowledge, *HEAL* is the first lncRNA that directly binds to the HIV promoter at the Nuc-0, HS, and Nuc-1 regions (Fig. 3A and B). We further demonstrated that *HEAL*-FUS formed an RNP complex to regulate HIV transcription (Fig. 3C to F). Here, the binding of FUS on the HIV promoter was dependent on *HEAL* at HS and Nuc-1 regions but not at the Nuc-0 region (Fig. 3F). One possibility is that as *HEAL* has different domains that function in binding the HIV promoter and/or interacting with FUS and that *HEAL* binding on genomic DNA at the Nuc-0 region interferes with the FUS-interacting domain or alters FUS conformation for the ChIP quantification.

**FIG 8:**
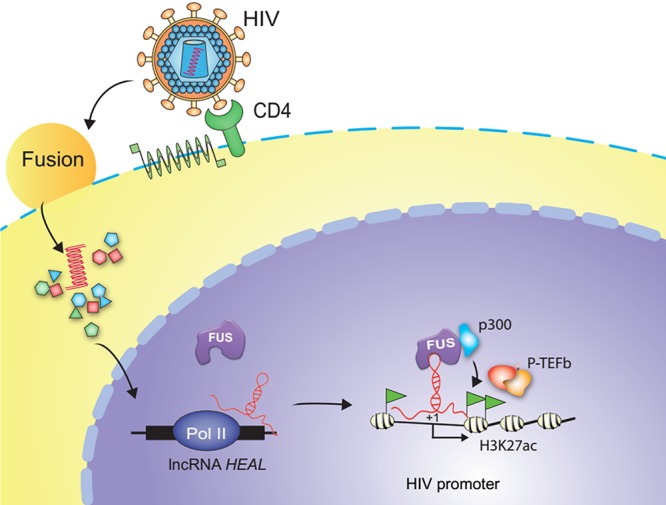
Proposed mechanism for *HEAL*- and FUS-mediated regulation of HIV-1 replication. HIV-1 infection enhances expression of *HEAL*, which interacts with FUS protein and increases *HEAL*-FUS binding at the HIV promoter. *HEAL*-FUS complex recruits histone acetyltransferase p300 to enhance H3K27ac modification, leading to P-TEFb enrichment on the HIV promoter and processive transcription. See the text for details.

Processive transcription from the HIV promoter is dependent on the function of P-TEFb that interacts with HIV Tat-TAR complex (37, 41, 43). P-TEFb is composed of two subunits, called cyclin T1 and CDK9 (37, 41, 43). Cyclin T1 binding near the TAR region was significantly decreased when *HEAL* expression was silenced (Fig. 4C). Together with the results that p300 as well as activation histone marker H3K27ac on HIV promoter was increased by *HEAL* (Fig. 4A and B) and that FUS interacts with HAT complex (39), we propose that *HEAL*-FUS complex was specifically enriched at the HIV promoter to enhance p300 recruitment and HIV transcription. Altogether, these results identified a new mechanism of an HIV-induced lncRNA, *HEAL*, that enhances HIV replication by directly binding to the HIV promoter and regulating its transcription by histone modification (Fig. 8).

*HEAL* regulates CDK2 expression in T cells through a direct interaction with the transcription factor FUS (Fig. 5). Increased CDK2 levels could enhance HIV-1 replication via several mechanisms. First, CDK2 has been shown to be an essential regulator of HIV-1 replication through phosphorylation and inactivation of SAMHD1, a phosphohydrolase that reduces the availability of deoxynucleoside triphosphates, thereby restricting HIV-1 replication. Inactivation of SAMHD1 thus increases the deoxynucleoside triphosphate pool available for HIV-1 reverse transcriptase and replication (44). Second, CDK2 phosphorylates serine 90 of CDK9, a component of the positive transcription elongation factor P-TEFb, supporting a role for CDK2 in the regulation of HIV-1 transcription (45). Third, efficient HIV-1 replication requires CDK2-mediated phosphorylation of a highly conserved threonine residue in the viral reverse transcriptase (46). CDK2-dependent phosphorylation thus enhances viral fitness by increasing the efficacy and stability of the reverse transcriptase (46). In addition, CDK2 can directly interact with FUS, as found in human embryonic stem cells (47), to potentially modulate FUS-mediated transcription and chromatin-remodeling functions. Our results showing that CDK2 expression is upregulated in HIV-1-infected patients further support an important role for this kinase in viral replication. This study thus identifies a new mechanism for HIV-1 regulation of CDK2 expression through enhanced transcription of the lncRNA *HEAL*.

We hypothesize that under ART, *HEAL* maintains a low level of expression that can be rapidly upregulated to reactivate viral replication when cells emerge from latency. *HEAL* expression targets the HIV promoter and enhances viral transcription by histone acetyl modification and elongation by P-TEFb. Our data showing that *HEAL* is elevated by HIV-1 infection in both MDMs and T cells demonstrated that targeting *HEAL* prevents viral recrudescence in both cell types when AZT is discontinued. Indeed, our data also suggest that inhibition of CDK2 is another potential strategy by which viral replication could be suppressed after ART cessation. Although no specific CDK2 inhibitors have been designed to date, targeting *HEAL* could indirectly inhibit CDK2 without influencing its other critical functions. Further studies are needed to define the precise signaling mechanism by which *HEAL* expression is induced after HIV-1 infection and how its expression is restricted during viral latency.

## MATERIALS AND METHODS

### Blood samples.

Peripheral blood from HIV-1-infected donors was collected into PAXgene tubes, and PBMCs were isolated as described below. RNA was isolated using TRIzol (Invitrogen) according to the manufacturer’s instructions. Parental or patient informed consent was obtained for all donors under a protocol approved by the Human Research Protection Program at UCSD.

### Cell culture.

MT4 and H9 cells were cultured in RPMI (Mediatech) containing 10% fetal bovine serum (FBS) and 50 μM β-mercaptoethanol (Sigma). E4 Jurkat cells were cultured in RPMI containing 10% FBS, penicillin-streptomycin, and 25 mM HEPES. MDMs were prepared by culture of PBMCs (see below) in RPMI containing 10% human serum for 1 week before seeding for experiments. 293FT cells were cultured in Dulbecco’s modified Eagle’s medium (DMEM) (Invitrogen) with 10% FBS.

### PBMC isolation.

A buffy coat fraction (25 ml) prepared by low-speed centrifugation of blood was centrifuged over Ficoll-Paque Plus (GE Healthcare) according to the manufacturer’s recommendations. The PBMC-containing interface layer was removed and washed with phosphate-buffered saline (PBS)–0.1% BSA followed by PBS. Cells were then collected and used for MDM preparation (above) or for RNA isolation.

### shRNA design and vector construction.

Sense and antisense sequences of shRNAs were designed using the open-access small interfering RNA (siRNA) selection program (48). shRNAs were obtained from Integrated DNA Technologies and cloned into the pLKO.1-puro lentiviral vector (Addgene) according to the manufacturer’s instructions. shRNA sequences are listed in Table S4 in the supplemental material. shRNAs targeting FUS were purchased from Thermo Fisher Scientific. For gene cloning, EGFP and *HEAL* were cloned into the pCDH-EF1-MCS lentiviral vector (System Biosciences; catalog no. CD502A-1) using the primers listed in Table S4.

### Lentivirus production and transduction.

293FT cells were seeded in 6-well plates at 8 × 10^5^/well 1 day before transfection. The culture medium was changed to Opti-DMEM (Life Technologies) before transfection. Lentiviral vectors were mixed with packaging vectors (System Biosciences) and transfected into 293FT cells using Lipofectamine 2000 (Invitrogen; catalog no. 11668019) according to the standard protocol for Opti-DMEM. The medium was replaced with DMEM containing 10% FBS 6 h after transfection. Two days after transfection, virus supernatants were collected and centrifuged at 4,000 rpm for 5 min to remove debris. For virus transduction, MT4 cells were seeded in 12-well plates at 4 × 10^5^ cells/well; infected by the addition of 500 μl virus supernatant, 4 μg/ml Polybrene, and 500 μl fresh medium; and then centrifuged at 750 rpm for 45 min at room temperature. The medium was refreshed the next day, and the cells were cultured for at least 3 days before analysis.

### HIV-1^LAI^ and HIV-1^BaL^ production and transduction.

293FT cells were seeded in 10-cm plates at 3 × 10^6^/well 1 day before transfection. The pLAI.2 or pBaL.01 vector was transfected into 293FT cells using Lipofectamine 2000 at a 1:2.5 ratio. Two days after transfection, virus supernatants were collected and centrifuged at 4,000 rpm for 5 min to remove debris. For HIV-1 transduction, MT4 or H9 cells were seeded in 12-well plates at 4 × 10^5^ cells/well and infected by the addition of 100 μl virus supernatant and 400 μl fresh medium for 4 h. The medium was refreshed, and the cells were cultured for at least 2 days (MT4) or 7 days (H9) before analysis.

### CRISPR-Cas9 editing of the *HEAL* locus.

The strategy to target *HEAL* exon2 with guide RNA is depicted in Fig. 6A. Briefly, guide sequences (*HEAL*, CCTCTTCCAGCCATTTATCCGTC; control, CGGAGGCTAAGCGTCGCAA) were generated as single-stranded oligonucleotides and annealed for cloning into lentiCRISPR v2 (Addgene 52961) according to previously published methods (34). Positive clones containing guides were identified by sequencing, and 1.8 μg of guide RNA vectors was transfected into 293FT cells to produce lentivirus. H9 cells were transduced with lentivirus, 1.0 μg/ml puromycin was added 24 h later, and the cells were cultured for 48 h. The surviving cells were cloned at 0.5 cells/100 μl/well in 96-well plates and cultured for 2 weeks. Single colonies were picked and screened using T7EI analysis (New England BioLabs [NEB]; catalog no. number E3321). Positive knockout clones were then sequenced to confirm biallelic modification of the *HEAL* locus.

### RNA extraction and RT-qPCR analysis.

Total RNA was extracted with TRIzol according to the manufacturer’s instructions. RNA was precipitated and dissolved in diethyl pyrocarbonate (DEPC)-treated water. Aliquots of 2 μg of total RNA were treated with Turbo DNase (Ambion) to remove contaminating genomic DNA, and the DNase-treated RNA was reverse transcribed using the iScript cDNA synthesis kit (Bio-Rad). qPCR was performed using 2× SYBR green mix (Bio-Rad) with cycling conditions of 95°C for 5 min followed by 50 cycles of 95°C for 10 s, 60°C for 10 s, and 72°C for 10 s. All qPCR primer sequences are listed in Table S4.

### lncRNA microarray design and analysis.

lncRNA microarrays were designed and prepared as described previously (31). Briefly, cDNA sequences of human lncRNAs were collected from two databases: 1,703 defined lncRNA sequences were downloaded from Ensembl (release 61) and 2,915 were downloaded from Havana (27). cDNA sequences were uploaded into the Agilent eArray custom microarray design system, and probes of 60 nucleotides in length were designed by the software. Probes with the potential for cross-hybridization were removed. In total, we obtained 12,281 probes for the 1,578 human lncRNA sequences from the Ensembl database and 15,947 probes for the 2,827 sequences from the Havana database. The probes were used to generate a custom microarray from Agilent. For quality control, ∼26,000 commercially available mRNA probes were also included in the array (Table S1).

### Rapid amplification of cDNA ends.

To deplete rRNA, total RNA isolated from MT4 cells was processed using a Ribo-Zero rRNA removal kit according to the manufacturer’s user manual (Epicentre). RACE was performed using a SMARTer RACE kit (Clontech) according to the manufacturer’s instructions. Briefly, ∼100 ng of purified poly(A) RNA was used for each RT-PCR. lncRNA cDNA samples (20 μl) were diluted ∼10-fold and used for nested PCRs. One primer was used for 3′ RACE, and two primers were used for 5′ RACE. Primer sequences are given in Table S4.

### Chromatin isolation by RNA purification.

MT4 cells (4 × 10^7^) were collected and fixed with 1% glutaraldehyde in 40 ml PBS for 10 min at room temperature, and the reaction was quenched by addition of 4 ml of 1.25 M glycine for 5 min at room temperature. The samples were centrifuged at 2,000 relative centrifugal force (RCF) for 5 min and washed twice with cold PBS, and the pellets were flash-frozen in liquid nitrogen and stored at −80°C. The pellets were thawed, mixed with 10 volumes of lysis buffer (50 mM Tris-HCl, pH 7.0, 10 mM EDTA, 1% SDS), and sonicated in a 4°C water bath at the highest setting for intervals of 30 s on and 45 s off for a total of 2 h. The cell lysate was centrifuged at 16,100 RCF for 10 min at 4°C, and the supernatant was collected. For the pulldown assay, the chromatin extract was mixed with 2 volumes of hybridization buffer and incubated with 2 μg biotinylated and folded *in vitro*-transcribed RNA for 4 h at 37°C with shaking. After hybridization, samples were incubated with 40 μl streptavidin-conjugated beads for 1 h with shaking, and the DNA and RNA were eluted from the beads as previously described (33, 34).

### Biotinylation of RNA and RNA-protein pulldown assays.

linc0492 and *lacZ* were cloned into pBluescript KSII downstream of the T7 promoter. The plasmid was linearized by single digestion with EcoRI, and 1 μg of linear RNA was transcribed and biotin labeled *in vitro* using an AmpliScribe-T7-Flash-biotin-RNA transcription kit (Epicentre) according to the manufacturer’s instructions. MT4 cells (4 × 10^7^) were resuspended in 4 ml of kit buffer A, and the RNA-protein pulldown assays were performed according to published methods (31).

### Chromatin immunoprecipitation.

Uninfected or HIV-1-infected H9 or MT4 cells (2 × 10^7^) were collected and fixed in 1% formaldehyde for 10 min at room temperature, and the reaction was quenched by addition of 125 mM glycine. Rabbit anti-FUS antibody (ab84078; Abcam) was used to immunoprecipitate endogenous FUS protein for ChIP assays. Anti-cyclin T1 antibody (81464; Cell Signaling Technology [CST]), anti-p300 (ab14984; Abcam), and anti-H3K27ac (ab4729; Abcam) were used to immunoprecipitate target proteins for ChIP assays. Rabbit IgG (2729; CST) was used as isotype control. and isotype control assays were performed according to published methods (49).

### Western blotting.

Cell lysates or immunoprecipitated products were separated by 10% SDS-PAGE and transferred to polyvinylidene difluoride (PVDF) membranes. Membranes were blocked with 5% nonfat milk in Tris-buffered saline containing 0.1% Tween 20 (TBST) and incubated with primary antibodies overnight at 4°C. Blots were washed and incubated with horseradish peroxidase (HRP)-conjugated secondary antibodies for 1 h at room temperature. Finally, blots were washed and visualized with ECL substrate (Pierce). Antibodies and dilutions were rabbit anti-HA (hemagglutinin)-tag monoclonal antibody (MAb) (C29F4) at 1:1,000 (Cell Signaling; 3724S), mouse anti-FLAG M2 antibody at 1:1,000 (Sigma-Aldrich; F1804), mouse anti-FUS MAb (4H11) at 1:200 (Santa Cruz; sc-47711), rabbit anti-CDK2 MAb (78B2) at 1:1,000 (Cell Signaling; S2546P), rabbit anti-DDX5 at 1:2,000 (Abcam; ab21696), and mouse antivimentin at 1:2,000 (Abcam; ab8978). Secondary antibodies were anti-mouse IgG (Santa Cruz; SC-2031) and anti-rabbit IgG (Pierce), both at 1:2,000.

### Statistical analysis.

Comparisons between two groups were analyzed using the Student *t* test. The differences were considered statistically significant when *P* was <0.05 (*, *P* < 0.05; **, *P* < 0.01; ***, *P* < 0.001; ****, *P* < 0.0001). Data are presented as mean ± SD.
