# Supplementary figures and images for: The Long Noncoding RNA HEAL Regulates HIV-1 Replication through Epigenetic Regulation of the HIV-1 Promoter
Source: mBio. 2019 Sep 24;10(5):e02016-19. doi: 10.1128/mBio.02016-19 (PMC6759764; doi:10.1128/mBio.02016-19)

Figure S1 (Related to Figure 2)

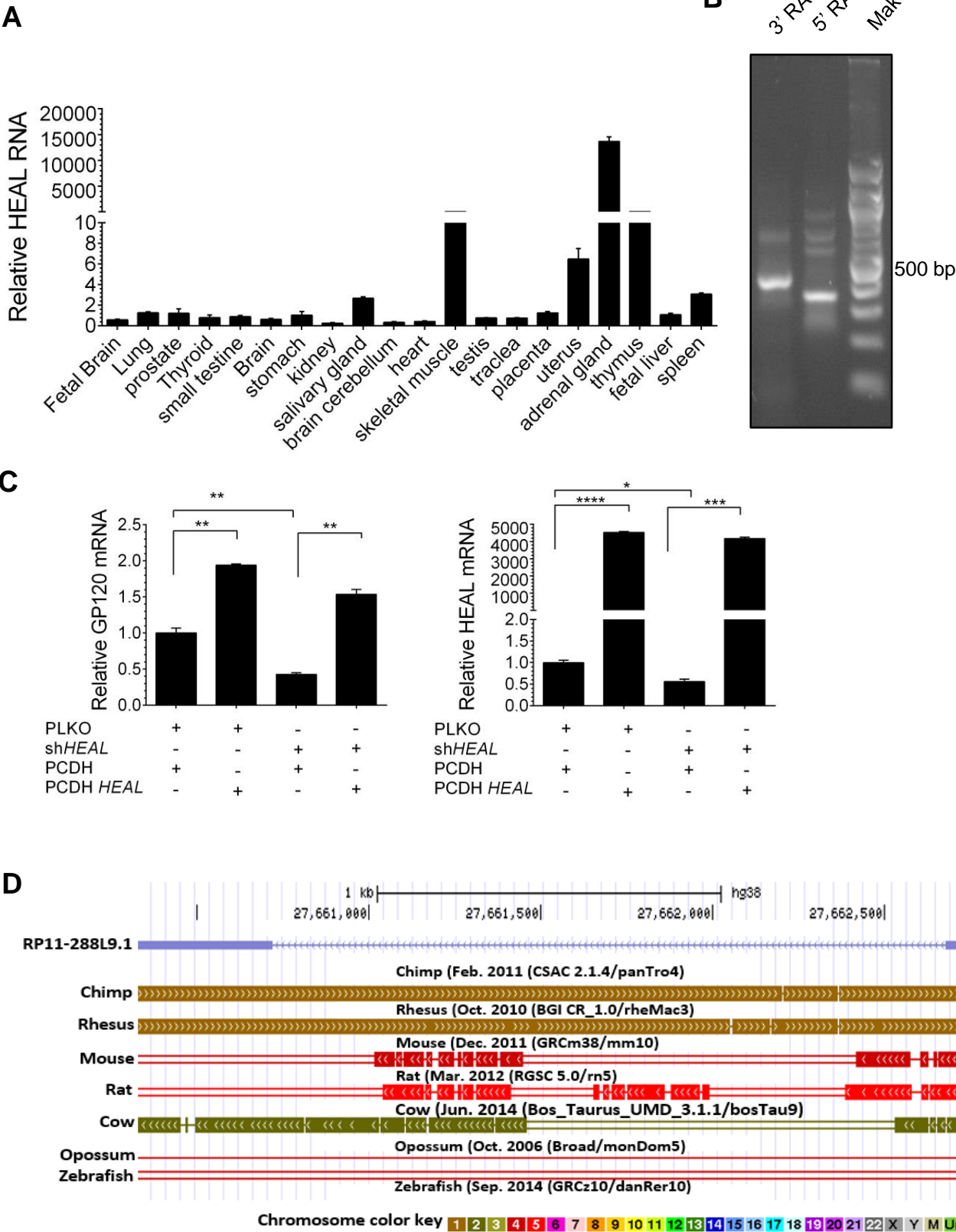

Supplement: FIG S1 [file mBio.02016-19-sf001.pdf]

Figure S2 (Related to Figure 5)

A

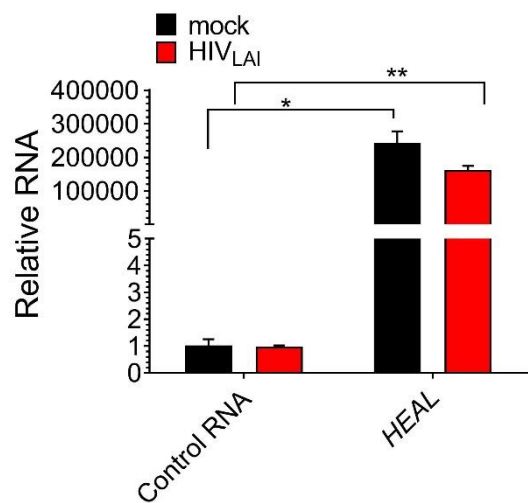

B

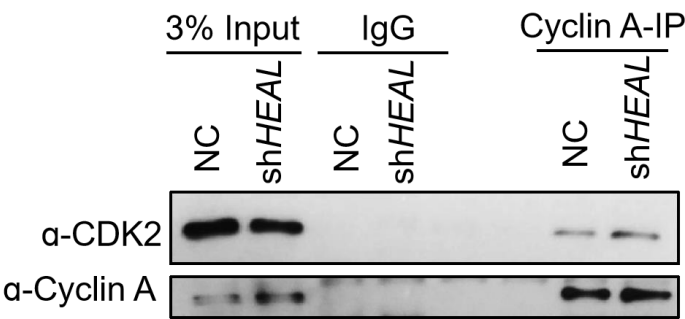

Supplement: FIG S2 [file mBio.02016-19-sf002.pdf]

Figure S3 (Related to Figure 6)

A

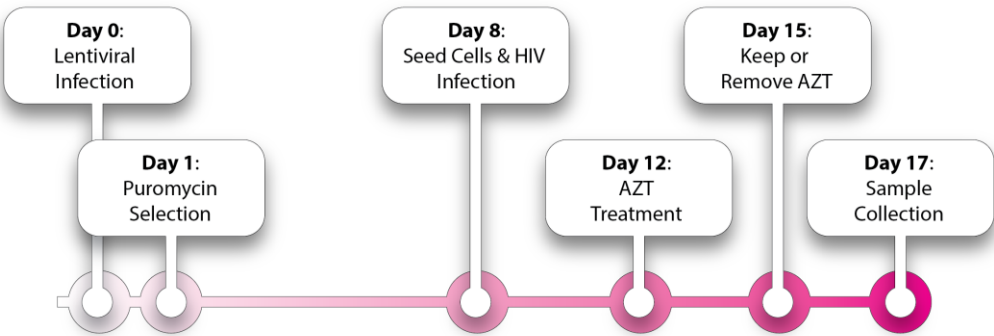

B

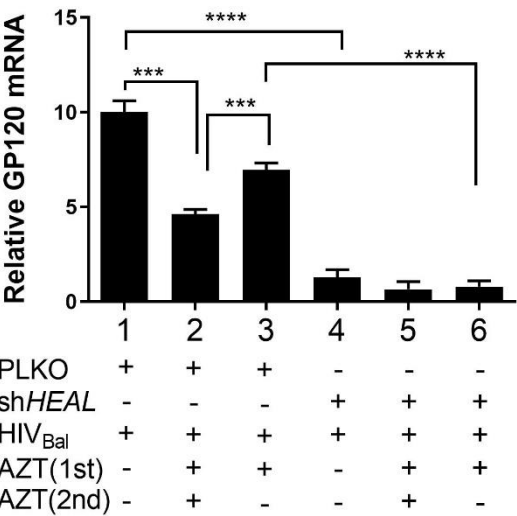

Supplement: FIG S3 [file mBio.02016-19-sf003.pdf]

**A**

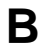

**C**

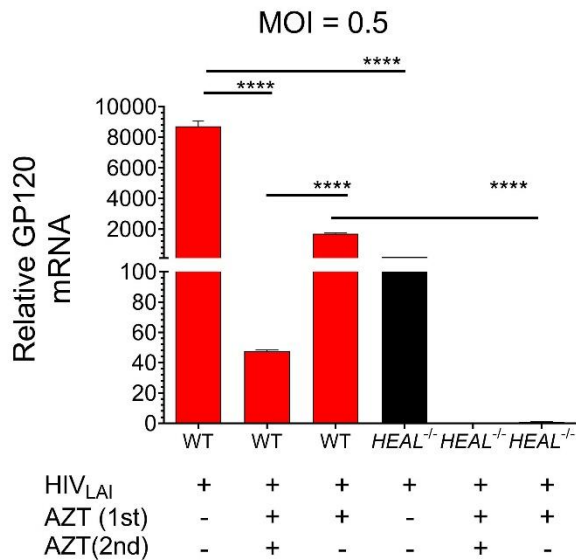

Supplement: FIG S4 [file mBio.02016-19-sf004.pdf]
