# Supplementary material for: The Long Noncoding RNA HEAL Regulates HIV-1 Replication through Epigenetic Regulation of the HIV-1 Promoter
Source: mBio. 2019 Sep 24;10(5):e02016-19. doi: 10.1128/mBio.02016-19 (PMC6759764; doi:10.1128/mBio.02016-19)
Supplement: TABLE S4 [file mBio.02016-19-st004.docx]

Table S4, related to Experimental Procedures (Real time qPCR and cloning). Oligonucleotides used in this study.

LinC0492 RACE primers:

| **Primer name** | **Sequence** |
| --- | --- |
| LinC0492GSP2 | CCCTCTGCCTGGAACACTTGTTGCTTCAG |
| LinC0492GSP1 | AGGGAGCTCATAATCTAGGGCGGAAGCC |
| LinC0492GSP1-B | GGCGGAAGCCTGATGGTAACAAATGCTC |

PCDH Primers:

| **Primer name** | **Sequence** |
| --- | --- |
| LinC0492 Forward: | AGT TCTAGA GACTCGCTGCTGTGC |
| LinC0492 Reverse: | AGT GAATTC GCCAGGGCATTTTAAA |
| EGFP Forward: | ATAGAATTCGCCACCATGGTGAGCAAGGGCGAGGA |
| EGFP Reverse: | ATAGCGGCCGCTCTAGATCCGGTGGATCCCG |

PLKO shRNA Primers:

| **Primer name** | **Sequence** |
| --- | --- |
| ENST00000440492 shRNA-1 F | CCGGAAGAGGGTTCATCTCTCTTTACTCGAGTAAAGAGAG  ATGAACCCTCTTTTTTTG |
| ENST00000440492 shRNA-1 R | AATTCAAAAAAAGAGGGTTCATCTCTCTTTACTCGAGTAAA  GAGAGATGAACCCTCTT |
| ENST00000440492 shRNA-2 F | CCGGAAGGGTTCATCTCTCTTTATACTCGAGTATAAAGAGAG  ATGAACCCTTTTTTTG |
| ENST00000440492 shRNA-2 R | AATTCAAAAAAAGGGTTCATCTCTCTTTATACTCGAGTATAAA  GAGAGATGAACCCTT |
| ENST00000440492 shRNA-3 F | CCGGAACCGTCCCAGAATGAATCATCTCGAGATGATTCATTCT  GGGACGGTTTTTTTG |
| ENST00000440492 shRNA-3 R | AATTCAAAAAAACCGTCCCAGAATGAATCATCTCGAGATGATT  CATTCTGGGACGGTT |
| ENST00000414116  shRNA-1 F | CCGGAAGGCCAAACATCTGGTTTGTCTCGAGACAAACCAGAT  GTTTGGCCTTTTTTTG |
| ENST00000414116  shRNA-1 R | AATTCAAAAAAAGGCCAAACATCTGGTTTGTCTCGAGACAAA  CCAGATGTTTGGCCTT |
| ENST00000414116  shRNA-2 F | CCGGAAGAGGCAAGATTGCGATATACTCGAGTATATCGCAATC  TTGCCTCTTTTTTTG |
| ENST00000414116  shRNA-2 R | AATTCAAAAAAAGAGGCAAGATTGCGATATACTCGAGTATATC  GCAATCTTGCCTCTT |
| ENST00000414116  shRNA-3 F | CCGGAAGGAGGCAAGATTGCGATATCTCGAGATATCGCAATC  TTGCCTCCTTTTTTTG |
| ENST00000414116  shRNA-3 R | AATTCAAAAAAAGGAGGCAAGATTGCGATATCTCGAGATATC  GCAATCTTGCCTCCTT |
| ENSG00000235304  shRNA-1 F | CCGGAAGTTGCCCACACTGGTAATACTCGAGTATTACCAGTGT  GGGCAACTTTTTTTG |
| ENSG00000235304  shRNA-1 R | AATTCAAAAAAAGTTGCCCACACTGGTAATACTCGAGTATTAC  CAGTGTGGGCAACTT |
| ENSG00000235304  shRNA-2 F | CCGGAAGATGGGAATTGTTGGATAACTCGAGTTATCCAACAAT  TCCCATCTTTTTTTG |
| ENSG00000235304  shRNA-2 R | AATTCAAAAAAAGATGGGAATTGTTGGATAACTCGAGTTATCC  AACAATTCCCATCTT |
| ENSG00000235304  shRNA-3 F | CCGGAACTCCAAGGCATGTTCATGACTCGAGTCATGAACATGC  CTTGGAGTTTTTTTG |
| ENSG00000235304  shRNA-3 R | AATTCAAAAAAACTCCAAGGCATGTTCATGACTCGAGTCATGA  ACATGCCTTGGAGTT |
| ENST00000417932  shRNA-1 F | CCGGAAGTGGCTCTTAGAGTGTAATCTCGAGATTACACTCTAA  GAGCCACTTTTTTTG |
| ENST00000417932  shRNA-1 R | AATTCAAAAAAAGTGGCTCTTAGAGTGTAATCTCGAGATTACA  CTCTAAGAGCCACTT |
| ENST00000417932  shRNA-2 F | CCGGAAGGAAGAGGGTATAGTTTATCTCGAGATAAACTATACC  CTCTTCCTTTTTTTG |
| ENST00000417932  shRNA-2 R | AATTCAAAAAAAGGAAGAGGGTATAGTTTATCTCGAGATAAAC  TATACCCTCTTCCTT |
| ENST00000417932  shRNA-3 F | CCGGAAGCACTCAACAAACCTACAACTCGAGTTGTAGGTTTGT  TGAGTGCTTTTTTTG |
| ENST00000417932  shRNA-3 R | AATTCAAAAAAAGCACTCAACAAACCTACAACTCGAGTTGTAG  GTTTGTTGAGTGCTT |

|  |
| --- |

qPCR primers:

| **Primer name** | **Sequence** |
| --- | --- |
| Linc0492 F | GTATCTCACCGTCCCAGAATG |
| Linc0492 R | GAGATGAACCCTCTGCTTGTC |
| Linc4116 F | GTGAGGCTAGTGTGAGGAGGA |
| Linc4116 R | GGCAGGTCACCGTTGAAA |
| Linc5304 F | TCAGCTGTCATTCTGCTGGT |
| Linc5304 R | TCCAGGGCCAAAGAACAA |
| Linc7932 F | GTGTAATTAGTCCAAGGGACAGC |
| Linc7932 R | GCTTTGGGATCACAACGATT |
| Linc8790 F | GCCTGATAGAATCTCACCACAA |
| Linc8790 R | CCAGAAGATTGAACCATGGGATA |
| GAPD F | ATGGGGAAGGTGAAGGTCG |
| GAPD R | GGGGTCATTGATGGCAACAAT |
| UBD F | AGAGATGGCTCCCAATGCT |
| UBD R | GGCATCAAAGGTCATTAAATCC |
| CDK2 F | TTAGTTGTTCAGTTGCCAAGGA |
| CDK2 R | ACTCCTATGGGGTAGGAGGTG |
| FCRL4 F | GCTGCACACAAACCTGTGAT |
| FCRL4 R | TCTCTCCTTTGAAGAATGTGGTC |
| PTK2B F | CAGCTCCGGAACGACTACAT |
| PTK2B R | ATTGTGGGGCATATCCTTGA |
| CCL3L1 F | CTGACTACTTTGAGACGAGCAG |
| CCL3L1 R | CTCCAGGTCACTGACGTATTTC |
| IFITM1 F | CACGCAGAAAACCACACTTC |
| IFITM1 R | TGTTCCTCCTTGTGCATCTTC |
| CT45A4 F | CTGCCATGTCCAAAGCAAAG |
| CT45A4 R | CAGGTTTCTGCATCATCCTATCT |
| CHST4 F | ACGTGTGGATGACCTTCAAG |
| CHST4 R | CACTGAAAGAGGCTGGACTG |
| DUSP1 F | TGGGTACATCAAGTCCATCTGA |
| DUSP1 R | GCAAAAAGAAACCGGATCAC |
| IL4I1 F | GCAAACCGAAAAGGATGACTG |
| IL4I1 R | TCCGGCTGTTGATCTTGATG |
| MAP3K8 F | CGCAAGAGGCTGCTGAGT |
| MAP3K8 R | TTCCTGTGCACGAAGAATCA |
| PITPNA F | ACATGGTGCTGCTCAAGG |
| PITPNA R | TGGCCTCAGCCACAGAATA |
| SUOX F | AACTACATCACACCCAACCC |
| SUOX R | GTGTAAGCGATAGGTGTCTGG |
| GP120 F (LAI) | TGA CAC AAT CAC ACT CCC ATG |
| GP120 R (LAI) | TCT AAT TTG TCC GCT GAT GGG |
| GAG F (Bal or 89.6) | TATCAGAAGGAGCCACCCCA |
| GAG R (Bal or 89.6) | CCCATTCTGCAGCTTCCTCA |
| IFI6 F | CAAGGTCTAGTGACGGAGCC |
| IFI6 R | GAGCAGCAGGTAGCACAAGA |

Promoter qPCR primers:

| **Primer name** | **Sequence** |
| --- | --- |
| CDK2 F | TAG CCC CCA AGA ACC AAA CT |
| CDK2 R | GGC CTT TCT ATT GGT CAA CG |
| UBD F | GGT TTG GGC TGT GGG TAT AG |
| UBD R | TGG GAT GGT CTT TCT GTG TTG |
| Nuc0-F | ATCTACCACACACAAGGCTAC |
| Nuc0-R | GTACTAGCTTGTAGCACCATCC |
| HS-F | TTACACCCTGTGAGCCTGCATG |
| HS-R | GCTCTCGGGCCACGTGAT |
| Nuc1-F | TCTCTGGCTAACTAGGGAACC |
| Nuc1-R | AAAGGGTCTGAGGGATCTCTAG |
| gGAPDH | CCAGCAAGAGCACAAGAGGA |
| gGAPDH | GAGATTCAGTGTGGTGGGGG |
